# IGF2BP2 modulates autophagy and serves as a prognostic marker in glioma

**DOI:** 10.1002/ibra.12150

**Published:** 2024-03-11

**Authors:** Ning Li, Limei Deng, Yuming Zhang, Xilian Tang, Bingxi Lei, Qingyu Zhang

**Affiliations:** ^1^ Department of Hematology Affiliated Hospital of Guangdong Medical University Zhanjiang Guangdong China; ^2^ The Marine Biomedical Research Institute Guangdong Medical University Zhanjiang China; ^3^ Department of Obstetrics and Gynecology Affiliated Hospital of Guangdong Medical University Zhanjiang Guangdong China; ^4^ Department of Neurosurgery, Sun Yat‐Sen Memorial Hospital Sun Yat‐Sen University Guangzhou China

**Keywords:** autophagy, glioma, IGF2BP2, LC3‐І/ІІ, p62

## Abstract

Glioma, a malignant brain tumor originating from neural glial cells, presents significant treatment challenges. However, the underlying mechanisms of glioma development are not fully understood, and effective targets are lacking. This study provides insights into the role of insulin‐like growth factor 2 messenger RNA‐binding protein 2 (IGF2BP2) in glioma progression and its therapeutic potential. Our analysis illustrated that elevated IGF2BP2 expression associated with significantly shorter survival among patients with low‐grade glioma (LGG) in The Cancer Genome Atlas (TCGA) database. IGF2BP2 depletion led to compromised cell viability, G0/G1 phase arrest, and reduced colony‐formation ability. Furthermore, ultrastructural analysis and mCherry‐GFP‐LC3 reporter assay revealed an increased abundance of autophagosomes upon IGF2BP2 knockdown. Western blot analysis corroborated these findings by showing reduced p62 levels coupled with increased LC3‐ІІ/LC3‐I ratio upon IGF2BP2 knockdown. A multicolor immunohistochemistry assay demonstrated the positive correlation between IGF2BP2 and p62 expression in glioma patient samples. Additionally, our analysis suggested a link between IGF2BP2 expression and drug‐resistant markers in TCGA‐LGG samples, and Cell Counting Kit‐8 cell viability assay revealed that knockdown of IGF2BP2 sensitized cells to temozolomide treatment. This comprehensive exploration unveils the role of IGF2BP2 in glioma progression, shedding light on autophagy modulation and chemosensitization strategies for glioma therapy.

## Graphical abstract

Depletion of insulin‐like growth factor 2 messenger RNA‐binding protein 2 in glioma cells demonstrates suppressed tumor growth, induction of autophagy, and heightened sensitivity to temozolomide treatment, offering a promising strategy to overcome resistance.

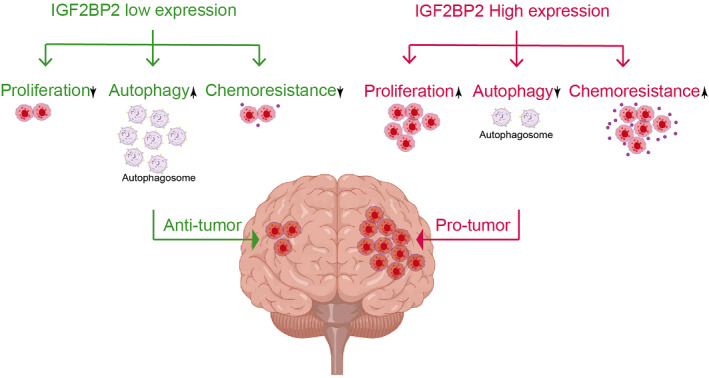

## 1 INTRODUCTION

Gliomas, a heterogeneous group of primary brain tumors originating from glial cells, present significant clinical challenges due to their diverse clinical manifestation and limited treatment options. Previously, the World Health Organization (WHO) classification categorized gliomas into a four‐grade system based on histological measurements of presumed malignancy,^1^ which served as the foundations of the modern classification of central nervous system tumors. The highest grade subtype is referred to as glioblastoma (GBM), known for its extreme aggressiveness and carries the poorest prognosis. Diffuse grade II and III gliomas are often referred to as diffuse low‐grade gliomas (LGGs) and typically exhibit lower invasiveness. However, this system faces challenges with interobserver variability and inconsistencies in pathological features and clinical outcomes.^2^ The updated versions of the WHO classification combined classical histopathology with diagnostic genetic features.^3^, ^4^ The incorporation of molecular markers such as isocitrate dehydrogenase (IDH) mutations, codeletion of chromosomal arms 1p and 19q (1p/19q codeletion), and telomerase reverse transcriptase promoter methylation has greatly enhanced the accuracy and reproducibility of diagnoses.^5^, ^6^ Currently, the standard treatments for gliomas include surgery, radiation therapy, and chemotherapy. However, more effective targeted therapies are urgent to be developed, especially for those patients with aggressive forms of the disease.^7^, ^8^, ^9^ Therefore, there remains a critical need for further research to identify and refine novel diagnostic and potential therapeutic molecular targets for gliomas.

The *N* ^*6*^‐methyladenosine (m6A) modifications are key regulators of posttranscriptional gene expression and cellular responses. These modifications are dynamically regulated by an intricate interplay of “writers,” “erasers,” and “readers”^10^ collectively influencing RNA translation, splicing, stability, and RNA–protein interactions.^11^, ^12^, ^13^, ^14^, ^15^, ^16^ Insulin‐like growth factor 2 (IGF2) messenger RNA (mRNA)‐binding protein 2 (IGF2BP2), a member of the m6A readers, was initially recognized for its role in IGF2 regulation. However, recent studies have unveiled its diverse functions, indicating its involvement in various cellular processes and diseases, including cancer.^17^, ^18^, ^19^ The emerging role of IGF2BP2 in glioma biology from several studies raises questions about its potential contribution and mechanism to glioma development and progression.^20^, ^21^, ^22^

Autophagy, a highly conserved cellular process involved in the degradation and recycling of cellular components, exhibits a dual role in cancer, acting as both a pro‐survival mechanism and a tumor suppressor.^23^, ^24^, ^25^, ^26^, ^27^, ^28^, ^29^ This complexity extends to gliomas, where autophagy's contextual influence plays a pivotal role in tumor progression.^26^, ^30^, ^31^ In certain instances, autophagy serves as a tumor suppressor in gliomas,^32^, ^33^ while in others, it is manipulated by gliomas to resist chemotherapies.^26^, ^34^, ^35^ Understanding this nuanced relationship between autophagy and glioma progression is crucial, offering valuable insights into potential therapeutic strategies. In this study, we identified IGF2BP2 as a poor prognostic marker among patients with LGG in The Cancer Genome Atlas (TCGA) database. Our study reveals a novel role for IGF2BP2 in autophagy regulation, as evidenced by the modulation of p62 and the ratio of LC3‐II/LC3‐I upon IGF2BP2 knockdown. The correlation between IGF2BP2 and p62 expression in patient tumor samples highlights the clinical relevance of our findings. This work contributes to our understanding of the complex interplay between autophagy and glioma progression, laying the groundwork for future investigations aimed at leveraging autophagy modulation as a therapeutic strategy in glioma treatment.

## 2 MATERIALS AND METHODS

### 2.1 TCGA database analysis

Gene expression profiles and corresponding clinical data were sourced from the Genomic Data Commons repository (https://portal.gdc.cancer.gov/) in level 3 HTSeq‐fragments per kilobase per million (FPKM) format. To standardize the assessment, FPKM values were transformed into transcripts per million reads. According to the expression of target genes (IGF2BP2 or p62) in patients with glioma, the data set was dichotomized into high and low expression cohorts using the median threshold. Rigorous statistical evaluation was conducted, including the Shapiro–Wilk *W* test and Wilcoxon rank‐sum test, with a significance level set at *p* < 0.05.

Key clinical parameters such as WHO grade, IDH status, and 1p/19q codeletion status were acquired from the comprehensive study by Ceccarelli et al.^36^ Similar to prior steps, the median threshold was used to segment data into high and low expression groups. Statistical analysis involved the Shapiro–Wilk normality test and the Wilcoxon rank‐sum test, with significance at *p* < 0.05.

To analyze survival outcomes, Kaplan–Meier (KM) curves were generated using the GEPIA 2 online tool (http://gepia2.cancer-pku.cn/#survival). The data set was divided into subsets of high and low expression using the median threshold. Statistically robust analysis was performed using the log‐rank test, with significance recognized at *p* < 0.05. Hazard ratios were assessed using the Cox proportional hazards model. The TISIDB online tool (http://cis.hku.hk/TISIDB/index.php) was utilized to assess the correlation between IGF2BP2 expression and overall survival in various human cancers.

To explore correlations between mRNA expression of IGF2BP2 and drug‐resistant markers, Pearson correlation was employed, quantifying associations using Pearson's correlation coefficient (*r*), with statistical significance denoted by *p* < 0.05.

### 2.2 Mammalian cell culture

Mammalian cell lines, including U87, HS683, and U251 cells, purchased from Xiamen Immocell Biotechnology company, were cultured in Dulbecco's modified Eagle medium supplemented with 10% fetal bovine serum, 2 mM glutamine, and 100 U/mL penicillin/streptomycin. The cells were maintained in a 37°C incubator with 5% CO_2_.

### 2.3 Knockdown of IGF2BP2 using short hairpin RNA (shRNA)

To achieve effective knockdown of IGF2BP2, two distinct shRNA sequences were employed. The sequences for these shRNAs were as follows: shIGF2BP2‐1: CCGGGGTGCCTGCAGCGGTAATATACTCGAGTATATTACCGCTGCAGGCACCTTTTTG; shIGF2BP2‐2: CCGGGCCGTTGTCAACGTCACATATCTCGAGATAT GTGACGTTGACAACGGCTTTTTG. A nonspecific control RNA sequence was employed as control (shNC RNA): GTTCTCCGAACGTGTCACGTCTCGAGACGTGACACGTT CGGAGAACTTTTT. The pLKO.1 vector harboring the respective shRNA or shNC RNA were cotransfected with the pCMV‐dR8.2 dvpr and pCMV‐VSV‐G plasmids in HEK 293T cells to produce lentivirus. After 40 h posttransfection, lentivirus was harvested and filtered through a 0.45 μm filter. The lentivirus was then used to infect the target cells (U87, HS683, or U251 cells). Following a 2‐day incubation period postinfection, the cells were subjected to selection using 2.5 μg/mL puromycin.

### 2.4 RNA isolation and real‐time quantitative polymerase chain reaction (RT‐qPCR)

Total RNA was isolated using the RNAeasy™ Animal RNA Isolation Kit (Cat# R0027; Beyotime) following the manufacturer's instructions. The HiScript Q RT SuperMix for qPCR kit (Cat# R223; Vazyme) was utilized following the manufacturer's protocol to synthesize complementary DNA. RT‐qPCR was performed using the ChamQ SYBR Color qPCR Master Mix (Cat# Q321; Vazyme) following the manufacturer's instructions (Tables 1 and 2). The primer sequences used for RT‐qPCR were as follows: IGF2BP2‐Forward: AGTGGGAGGTGTTGGATGG; IGF2BP2‐Reverse: CGGTTTCTGTGTCTGTGTTG; GAPDH‐Forward: GTCTCCTCTGACTTCAACAGCG; GAPDH‐Reverse: ACCACCCTGTTGCTGTAGCCAA. To quantify the relative mRNA expression levels, the established $2^{-∆∆C_{t}}$ method was applied.^37^

**Table 1:** The reagents of reaction mixture for RT‐qPCR.

Reagent name	Volume (µL)
2 × ChamQ SYBR qPCR Master Mix (without ROX)	10.0
Forward primer (10 µM)	0.4
Revierse primer (10 µM)	0.4
Template DNA/cDNA	1.0
ddH_2_O	To 20.0

Abbreviations: cDNA, complementary DNA; RT‐qPCR, real‐time quantitative polymerase chain reaction.

**Table 2:** The procedure of RT‐PCR.

Stage	Name	Cycle	Temperature (°C)	Time (s)
Stage 1	Initial denaturation	Rep: 1	95	30
Stage 2	Cycling reaction	Reps: 40	95	10
			60	30
Stage 3	Melt curve	Rep: 1	95	15
			60	60
			95	15

Abbreviation: RT‐qPCR, real‐time quantitative polymerase chain reaction.

### 2.5 Cell Counting Kit‐8 (CCK8) assay

Cell viability was evaluated using the CCK8 (Cat# C0038; Beyotime). U87, HS683, and U251 cells with or without IGF2BP2 shRNA were seeded into 96‐well plates at a density of 2000 cells/well. The CCK8 reagent was added to the cells at different time slots (Days 1–3) with a 24‐h interval, followed by an incubation period of 1 h at 37°C. The absorbance was measured at a wavelength of 450 nm using a microplate reader (Agilent BioTek Epoch Microplate Spectrophotometer). The absorbance values were corrected by subtracting the background absorbance measured from wells containing only the culture medium and CCK8 solution without cells. The corrected absorbance values were then used to calculate the percentage of viable cells compared to control wells using the following formula: cell viability = absorbance of treated sample/absorbance of control sample.

### 2.6 Cell cycle analysis

U87, HS683, and U251 cells with or without IGF2BP2 shRNA were seeded into six‐well plates at an appropriate density (5 × 10^5^/mL). Afterward, the cells were trypsinized, rinsed with phosphate‐buffered saline (PBS), and fixed in 70% ethanol at 4°C overnight. Subsequently, the fixed cells were collected, PBS‐washed, and then stained with propidium iodide (PI) from the Cell Cycle and Apoptosis Analysis Kit (Cat# BL114A; Biosharp) according to the manufacturer's instructions. The stained cells were subjected to analysis using a flow cytometer (BD FACSCanto™). To begin, cell populations were selected based on their forward scatter and side scatter characteristics to exclude cellular debris and ensure intact cell inclusion. Following this, the cells were gated using PI fluorescence signals (PI‐A and PI‐W) to assess DNA content. The distribution of cells in different phases of the cell cycle was determined through the utilization of the Sync Wizard project in MODFIT software (ModFit LT™).

### 2.7 Colony‐formation analysis

To assess the colony‐forming ability of cells subjected to IGF2BP2 shRNA, a colony‐formation assay was executed. U87, HS683, and U251 cells with or without IGF2BP2 shRNA were cultured in six‐well plates, initiated at a density of 200 cells/mL. Cells were cultured in a 37°C incubator until the control cells with shNC develop visibly distinct colonies. Subsequently, the cells were fixed with 4% paraformaldehyde and stained with 0.5% crystal violet in 20% ethanol. The plates were then undergoing gentle rinsing with tap water, followed by drying at room temperature. Colonies were visually counted to evaluate the impact of IGF2BP2 knockdown on the clonogenic potential of the glioma cells.

### 2.8 Transmission electron microscopy (TME) ultrastructural analysis

U87 cells with or without IGF2BP2 knockdown were fixed with 3% glutaraldehyde. Subsequently, the cells were postfixed in osmium tetroxide and dehydrated using a series of ethanol (30%, 50%, 70%, 90%, and 100%). The dehydrated cells were embedded by infiltrating with epoxy resin and cut to ultrathin sections. Finally, TEM was used to visualize the internal structures of cells and capture images.

### 2.9 mCherry‐GFP‐LC3 reporter assay

U251 cells stably expressing the mCherry‐GFP‐LC3 reporter construct were subjected to lentivirus transduction to knockdown IGF2BP2 using shRNA. In the mCherry‐GFP‐LC3 reporter assay, the green fluorescent spots of GFP, which are sensitive to the acidic environment of lysosomes, indicate the presence of autophagosomes, while the red fluorescent spots of mCherry, being more stable, signify autolysosomes. Following the knockdown of IGF2BP2, cells were fixed using 4% paraformaldehyde and mounted using Antifade Mounting Medium with Hoechst 33342 (Cat# C1025; Beyotime) for nuclear staining. Subsequently, confocal microscopy (Olympus IXplore SpinSR Super Resolution Microscope) was employed to capture fluorescent images of the stained cells. The fluorescent spots were then quantified and analyzed to assess the generation of autophagosomes and autolysosomes.

### 2.10 Western blot

The cells were lysed using cell lysis buffer for Western and immunoprecipitation analysis (Cat# P0013; Beyotime) to extract cellular proteins. The concentration of proteins in the cell lysate was determined using the Bradford Protein Assay Kit (Cat# P0006C; Beyotime). Equal amounts of protein from cell lysates were loaded onto a 12% sodium dodecyl sulfate gel and subsequently transferred onto a polyvinylidene fluoride membrane. The membrane was incubated with specific primary antibodies at 4°C overnight, including anti‐IGF2BP2 (Cat# YT2284, 1:1000; Immunoway), anti‐β‐Actin (Cat# 66009‐1‐Ig, 1:1000; Proteintech), anti‐p62 (Cat# 18420‐1‐AP, 1:5000; Proteintech), and anti‐LC3 (Cat# 12741, 1:1000; Cell Signaling Technology). After incubation with primary antibodies, the membrane was washed with Tris‐buffered saline with 0.1% Tween‐200 (TBST) and incubated with suitable secondary antibodies, horseradish peroxidase (HRP) goat anti‐mouse immunoglobulin G (IgG) (Cat# RS0001, 1:5000; Immunoway), or HRP‐labeled goat anti‐rabbit IgG (Cat# LF102, 1:5000; EpiZyme), at room temperature for 1 h. To detect the presence of HRP‐labeled antibodies, the MCE Ultra High Sensitivity ECL Kit (Cat# HY‐K1005; MedChem Express) was used. The results were visualized and captured using the GelView 1500 Pro II imaging system (BLT‐IMAGING).

### 2.11 Durg treatment

To evaluate the impact of IGF2BP2 on autophagy, U87 and U251 cells with IGF2BP2 shRNA were subjected to treatment with either 3‐methyladenine (3‐MA, 10 mM; Sigma‐Aldrich) or hydroxychloroquine (HCQ, 10 μM; Sigma‐Aldrich) for varying durations (1, 2, and 3 days). Besides, U87 and U251 cells with shNC were subjected to treatment with dimethyl sulfoxide. To investigate the role of IGF2BP2 in glioma cell sensitivity to temozolomide (TMZ; Beyotime) treatment, U87 and U251 cells with or without IGF2BP2 shRNA were exposed to a range of TMZ concentrations (0, 125, 250, 500, and 1000 μM). Cell viability assays were conducted using the CCK8 reagent to assess the effects of these treatments on cell viability.

### 2.12 Multicolor immunohistochemistry (mIHC)

The mIHC assay was conducted on a tissue microarray (TMA) chip containing tumor samples from 60 patients with clinical glioma at Sun Yat‐Sen Memorial Hospital, Sun Yat‐Sen University. The TMA construction protocol followed standard procedures as described previously.^38^ Briefly, representative areas of tumor tissues were identified on hematoxylin and eosin‐stained slides by a pathologist. Tissue cores with a diameter of typically 1–2 mm were then punched from the corresponding paraffin‐embedded tumor blocks and transferred to a recipient paraffin block. The TMA block was then heated to allow for the proper fusion of the tissue cores with the recipient block. Multiple sections (typically 4–5 μm thick) were cut from the TMA block using a microtome and mounted onto positively charged glass slides. The mIHC protocol utilized the four‐color multiple fluorescent immunohistochemical staining kit (Cat# abs50012; Absin) in accordance with the manufacturer's instructions. To prepare the TMA slide for staining, it was initially immersed in a xylene bath to remove paraffin, followed by sequential rehydration in fresh 100%, 95%, and 70% ethanol solutions. Subsequently, the slide was placed within a microwave‐resistant plastic staining jar, where it underwent antigen retrieval using antigen retrieval solution. After blocking with 5% bovine serum albumin, the slide was incubated at room temperature with the primary antibody anti‐IGF2BP2 (Cat# YT2284, 1:100; Immunoway) for 30 min, followed by three washes with TBST. Afterward, the slide was exposed to an HRP‐labeled secondary antibody. Subsequently, the tyramide signal amplification fluorescent dye was used for staining. The immunofluorescence staining procedure was then repeated for the primary antibody anti‐p62 (Cat# 18420‐1‐AP, 1:50; Proteintech) and the corresponding fluorescent dye staining. After the dual primary antibody and fluorescent dye incubation steps, the slide underwent 4′, 6‐diamidino‐2‐phenylindole staining to label cell nuclei. The stained TMA slide was scanned using the Pannoramic MIDI II system (3DHISTECH), and images were captured with SlideViewer software (3DHISTECH). Quantification of fluorescent intensity was performed using Image‐Pro Plus.

### 2.13 Statistical analysis

The homogeneity of variances among multiple groups was assessed using statistical tests to ensure the validity of subsequent analyses. First, Levene's test, Bartlett's test, and the Brown–Forsythe test were employed to evaluate whether the variances across groups were approximately equal. In cases where variances were found to be unequal, data transformation techniques (logarithmic and square root transformations) were applied to achieve homogeneity. The presented data were expressed as means ± standard deviation (SD). The statistical analysis was conducted using multiple *t* tests or paired two‐tailed *t* test, with significance levels denoted as follows: *p < 0.05; **p < 0.01; ***p < 0.001.

### 2.14 Ethics statement

The utilization of human glioma tumor TMA in our study was conducted in strict accordance with the regulations governing human genetic resource management in China. Ethical oversight and approval were secured through a comprehensive review process by the Medical Ethics Committee of Sun Yat‐sen Memorial Hospital, Sun Yat‐sen University (Protocol: SYSKY‐2023‐108‐01). The procedures involving human glioma tumor tissue microarray were performed in accordance with the Declaration of Helsinki https://www.wma.net/what-we-do/medical-ethics/declaration-of-helsinki/). Informed consent was obtained from all patients who directly participated in this study.

## 3 RESULTS

### 3.1 Prognostic significance of IGF2BP2 overexpression in patients with TCGA‐LGG 

**Figure 1:**
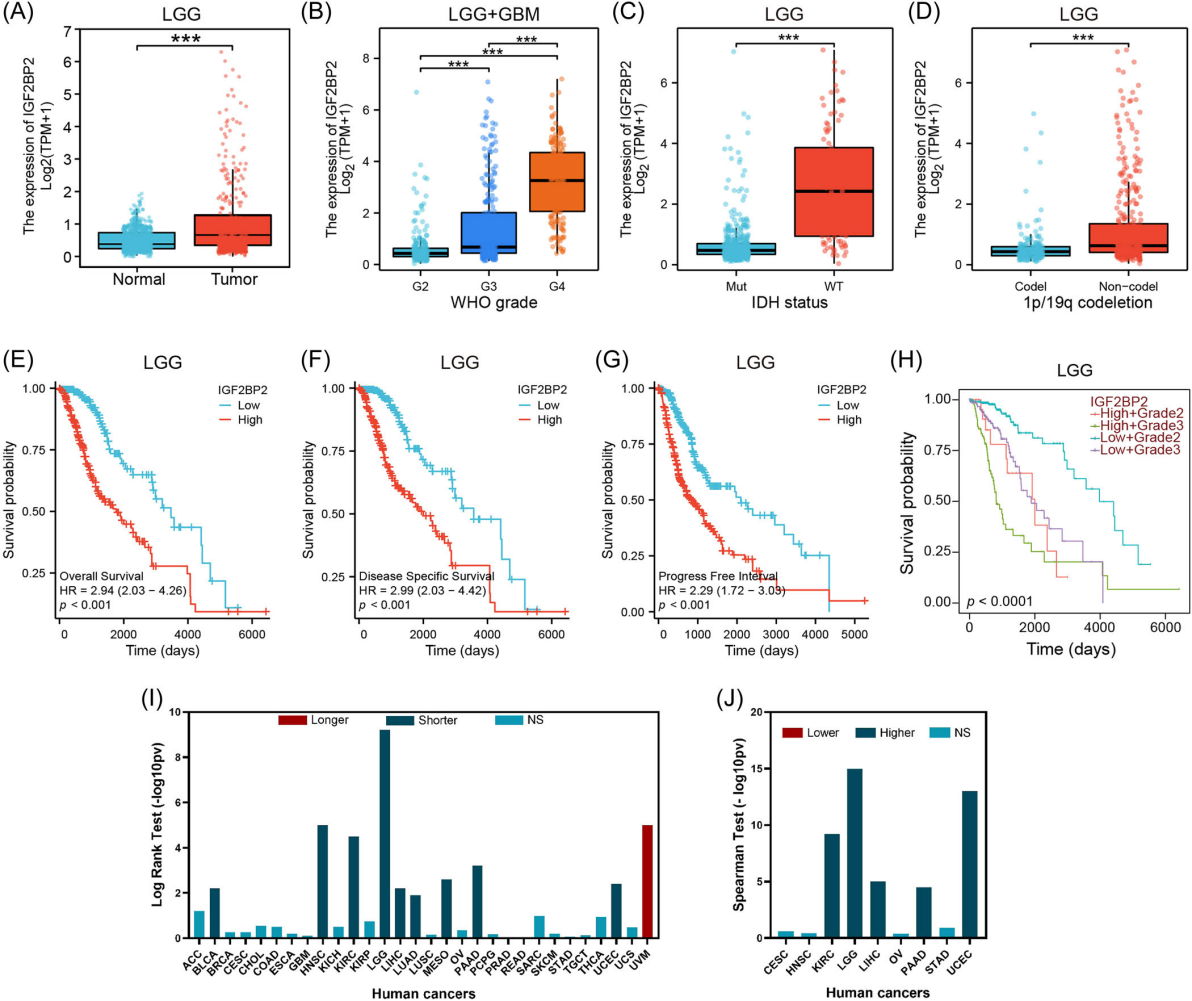
Correlation between expression of insulin‐like growth factor 2 messenger RNA‐binding protein 2 (IGF2BP2) and prognostic significance or clinical features in patients with TCGA‐low‐grade glioma (LGG). (A) Expression distribution of IGF2BP2 between tumor and normal tissues in patients with LGG. (B) Expression distribution of IGF2BP2 in different grades of patients with LGG and glioblastoma (GBM). (C) Expression distribution of IGF2BP2 in patients with isocitrate dehydrogenase (IDH)‐mutant or IDH wild‐type LGG. (D) Expression distribution of IGF2BP2 in patients with 1p/19q codeletion and 1p/19q non‐codeletion LGG. Kaplan–Meier curves showing overall survival (E), disease‐specific survival (F), progress‐free interval survival (G), and grade 2 or grade 3 overall survival (H) for patients with LGG with high and low expression of IGF2BP2. (I) The correlation between the expression of IGF2BP2 and overall survival across various human cancers. Red signifies a longer survival time, blue represents a shorter survival time, and cyan indicates no significant difference in survival outcomes. (J) Associations between the expression levels of IGF2BP2 and tumor grade across various human cancers. Red indicates that gene expression is associated with lower grade, blue implies an association with higher grade, and cyan denotes no significant association between gene expression and tumor grade. Cancer names are defined using their TCGA abbreviations (https://gdc.cancer.gov/resources-tcga-users/tcga-code-tables/tcga-study-abbreviations). HR, hazard ratio; NS, no significant. ***p < 0.001, **p < 0.01, *p < 0.05. TCGA, The Cancer Genome Atlas. [Color figure can be viewed at wileyonlinelibrary.com]

Exploration of IGF2BP2 expression within glioma was conducted through a comprehensive analysis using the TCGA program database. A significant upregulation of IGF2BP2 expression was observed in TCGA‐LGG tumors compared to normal tissues (Figure 1A). Additionally, within both TCGA‐LGG and TCGA‐GBM samples, higher grades of glioma correlated with a higher IGF2BP2 expression, suggesting its involvement in the malignant transformation of glioma cells (Figure 1B). The IDH wild‐type status and a 1p/19q noncodeletion status are typically more aggressive and associated with a poorer prognosis compared to gliomas with IDH mutations and 1p/19q codeletion.^4^, ^5^, ^6^, ^39^ Our results revealed that IGF2BP2 expression was notably higher in IDH wild‐type (Figure 1C) and 1p/19q noncodeletion cases (Figure 1D). The survival analysis, as depicted by KM curves, illuminated a significant connection: heightened IGF2BP2 expression was unequivocally associated with markedly reduced overall survival, disease‐specific survival, and progression‐free intervals among patients with TCGA‐LGG (Figure 1E–G). This relationship persisted when examining survival outcomes distinctly within grade 2 and grade 3 gliomas, where higher expression of IGF2BP2 predicated shorter survival times (Figure 1H). Pan‐cancer analysis similarly indicated that elevated IGF2BP2 expression correlated with shorter survival time (Figure 1I) and higher tumor grades (Figure 1J) across a diverse range of cancer types. These analyses highlighted IGF2BP2 as a potential prognostic marker for patients with glioma. Further investigation into the molecular mechanisms underlying the role of IGF2BP2 in glioma is warranted.

### 3.2 Knockdown of IGF2BP2 inhibited cell viability in glioma cells

**Figure 2:**
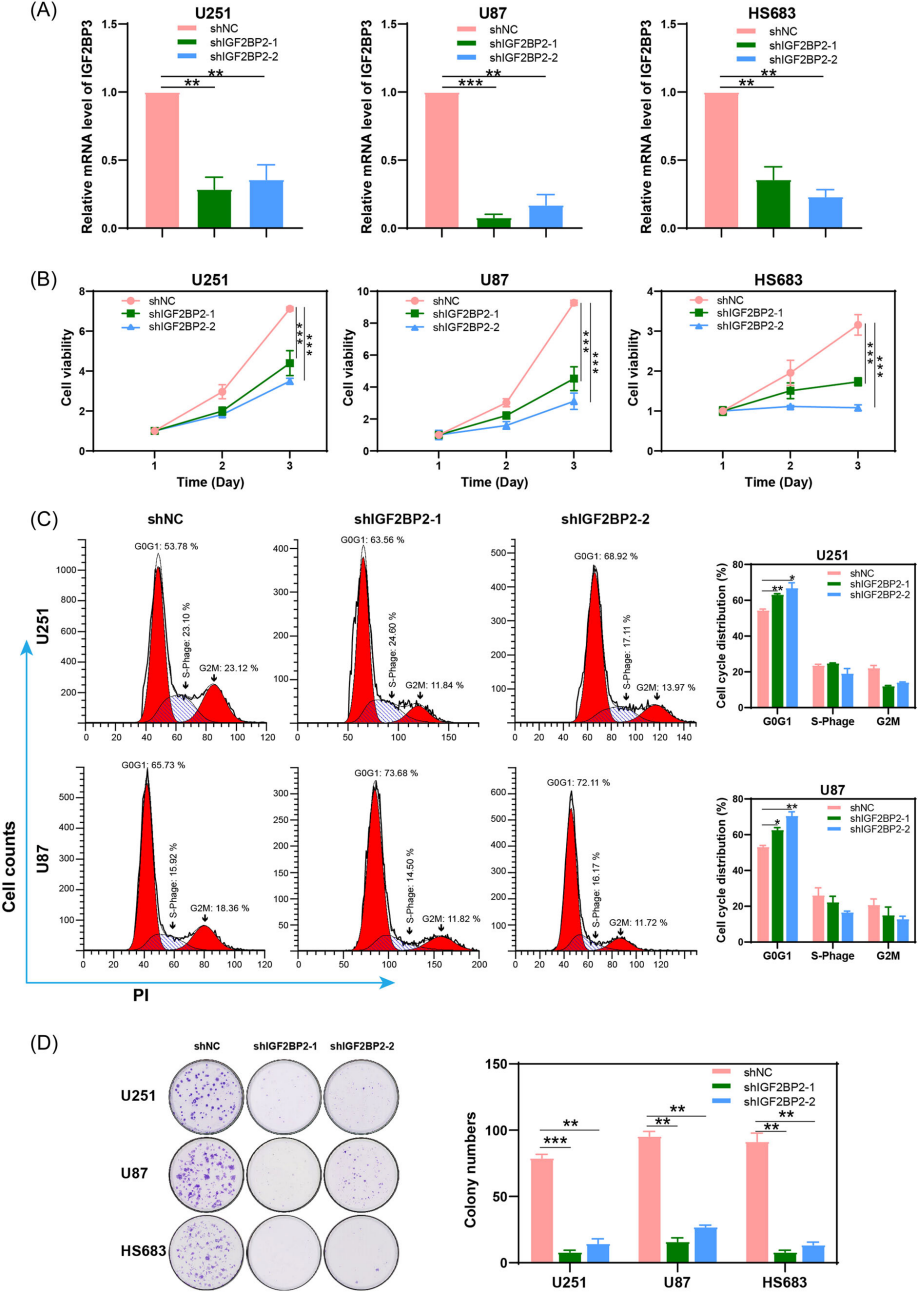
Knockdown of insulin‐like growth factor 2 messenger RNA (mRNA)‐binding protein 2 (IGF2BP2) resulted in impaired cell growth in glioma cells. (A) U87, U251, and HS683 cells were infected with short hairpin RNA lentivirus to knockdown IGF2BP2, while shNC lentivirus served as a control. Real‐time quantitative polymerase chain reaction was conducted to assess the efficiency of IGF2BP2 knockdown. (B) Cell Counting Kit‐8 assay indicates a notable reduction in cell viability in U87, U251, and HS683 cells following IGF2BP2 knockdown. (C) Cell cycle analysis of U251 and U87 cells with or without IGF2BP2 knockdown. (D) Colony‐formation assay showing effects of IGF2BP2 knockdown on clonogenicity in U87, U251, and HS683 cells. Data are presented as the mean ± standard deviation from three independent experiments. ***p < 0.001, **p < 0.01, *p < 0.05. [Color figure can be viewed at wileyonlinelibrary.com]

To investigate the functional role of IGF2BP2 in glioma, we employed shRNA to knockdown IGF2BP2 expression in glioma cells, including HS683, U87, and U251. A nonspecific scramble RNA was used as a shRNA control. The knockdown efficiency was validated by RT‐qPCR (Figure 2A). CCK8 assays demonstrated that IGF2BP2 knockdown led to impaired cell viability (Figure 2B). Moreover, the perturbation of IGF2BP2 levels induced a notable G0/G1 phase arrest within the cell cycle (Figure 2C). The role of IGF2BP2 in cancer malignancy was further accentuated by colony‐formation assays, where a significant reduction in the ability to form colonies was observed (Figure 2D). These findings collectively highlight the importance of IGF2BP2 in orchestrating glioma cell viability and proliferation.

### 3.3 Silencing IGF2BP2‐induced autophagy in glioma cells

**Figure 3:**
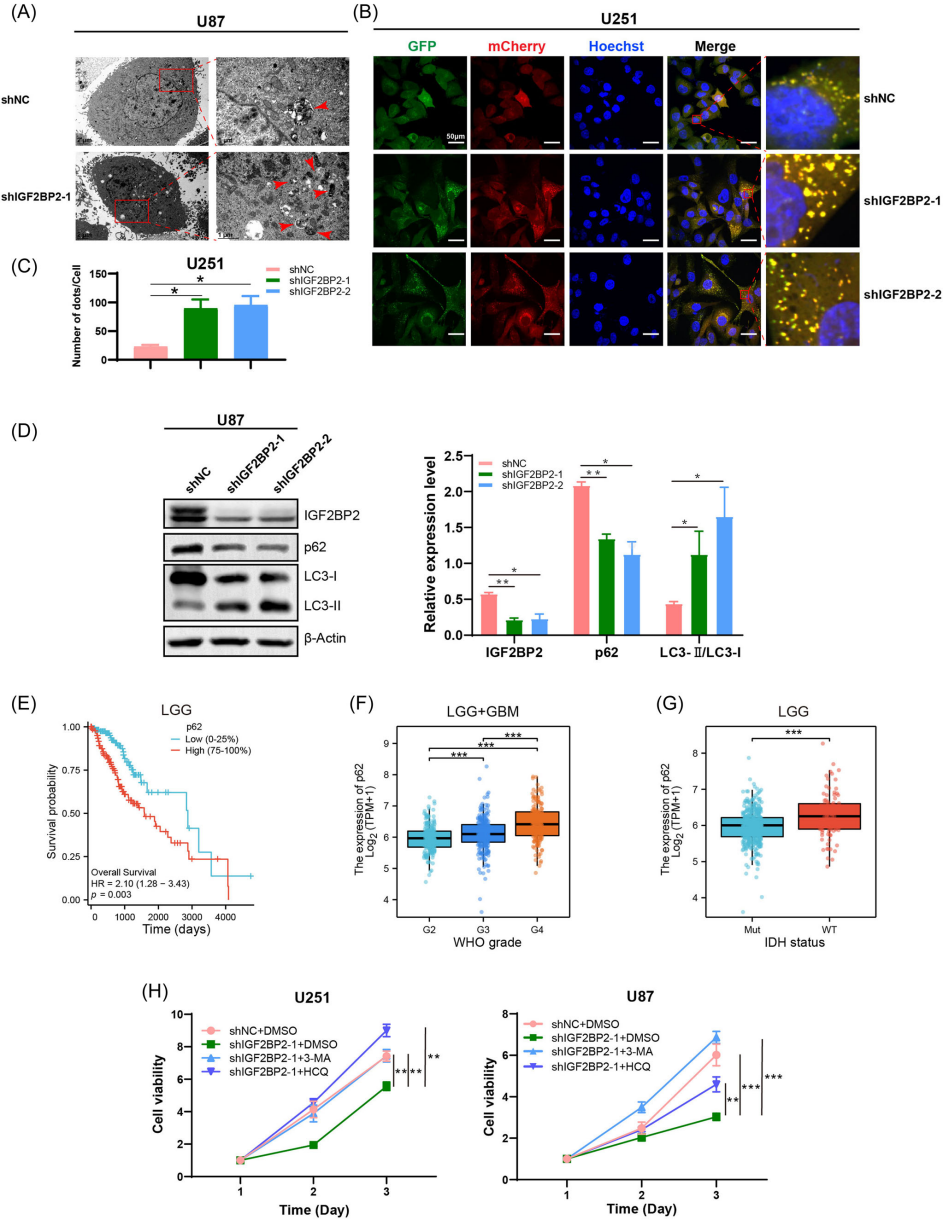
Insulin‐like growth factor 2 messenger RNA‐binding protein 2 (IGF2BP2) knockdown led to the activation of autophagy in glioma cells. (A) Transmission electron microscopy images revealed that IGF2BP2 knockdown in U87 cells resulted in an increased presence of autophagosomes and autolysosomes, as indicated by the red arrows. (B) mCherry‐green fluorescent protein (GFP)‐LC3 reporter assay demonstrated that IGF2BP2‐KD U251 cells exhibited more autophagosomes and autolysosomes compared to the control cells. The green fluorescent indicates GFP‐positive cells (autophagosomes), the red fluorescent indicates mCherry (autolysosomes), and blue indicates Hoechst (nuclear). Scale bar = 50 μm. (C) Quantification of fluorescent spots in (B). (D) Western blot detecting the expression of p62 and LC3‐I/II after IGF2BP2 knockdown in U87 cells. (E) Kaplan–Meier curves showing overall survival for patients with low‐grade glioma (LGG) with high and low expression of p62. (F) Expression distribution of p62 in different grades of patients with LGG and glioblastoma. (G) Expression distribution of p62 in patients with isocitrate dehydrogenase (IDH) mutant or IDH wild‐type LGG. (H) A Cell Counting Kit‐8 assay was then performed to assess the impact of these autophagy inhibitors, 3‐methyladenine, and hydroxychloroquine (HCQ), on the viability of IGF2BP2‐KD cells.  Data are presented as the mean ± standard deviation (SD) from three independent experiments. ***p < 0.001, **p < 0.01, *p < 0.05. [Color figure can be viewed at wileyonlinelibrary.com]

**Figure 4:**
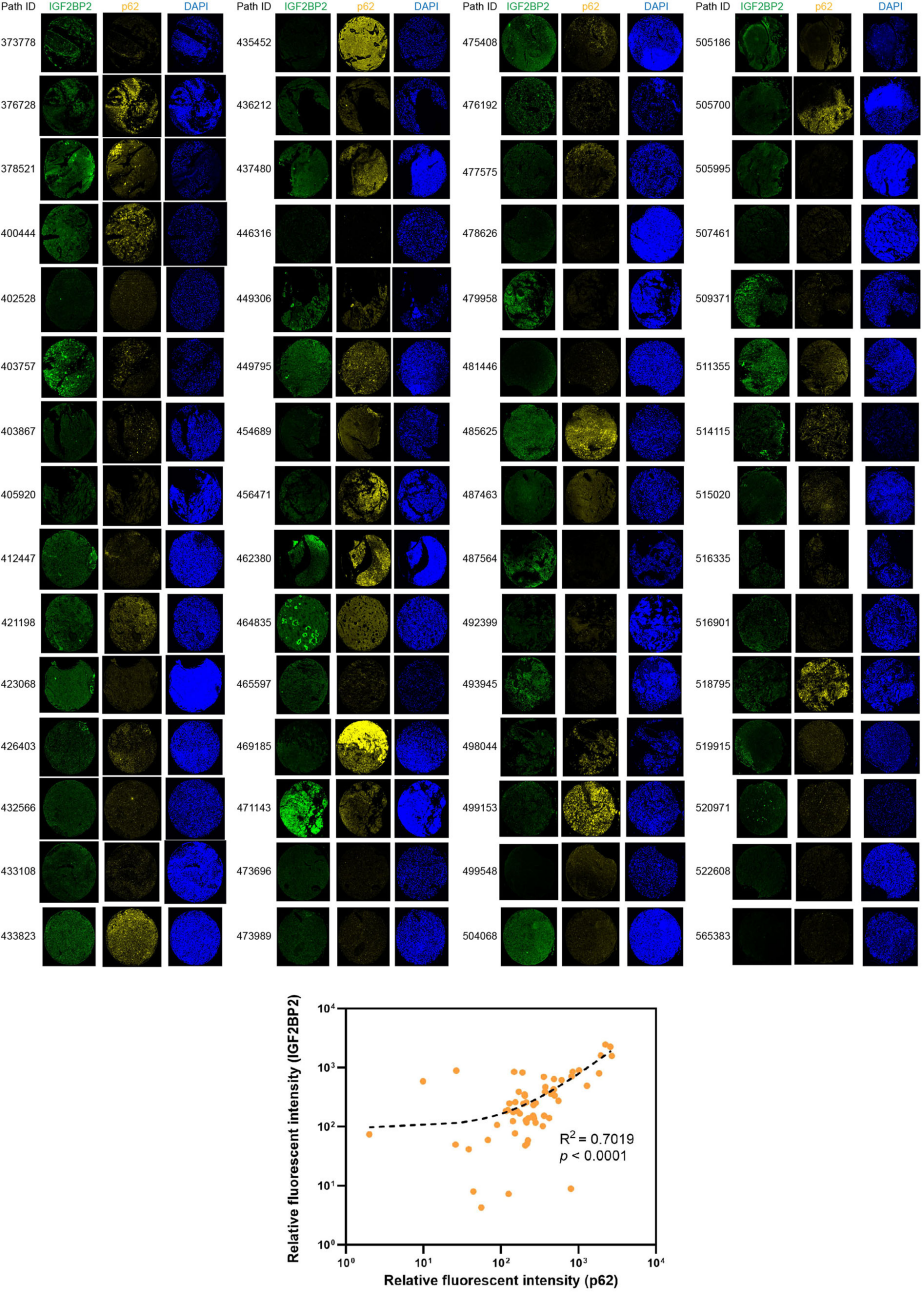
The multicolor Immunohistochemistry (mIHC) analysis of insulin‐like growth factor 2 messenger RNA‐binding protein 2 (IGF2BP2) and p62 expression in glioma tissue microarray (TMA) chip. The mIHC was conducted to examine the expression of IGF2BP2 and p62 in a TMA Chip. Green represented IGF2BP2, yellow represented p62, and blue represented 4′, 6‐diamidino‐2‐phenylindole. Scale bar = 50 μm. This TMA chip encompassed 60 tumor samples from patients with clinical glioma. The fluorescent intensity of the staining was indicative of the respective protein expression levels, and the quantification was performed using Image‐Pro Plus software. A linear regression approach was utilized to establish the correlation between the expression levels of IGF2BP2 and p62. [Color figure can be viewed at wileyonlinelibrary.com]

Upon IGF2BP2 knockdown, U87 cells exhibited an augmented formation of autophagosomes, as evidenced by TME (Figure 3A). This observation gained further support from a mCherry‐GFP‐LC3 reporter assay, which showed a substantial increase in the accumulation of LC3 puncta in U251 IGF2BP2 knockdown cells (Figure 3B,C). Moreover, western blot analysis demonstrated a decrease in p62 expression coupled with increased LC3‐II/LC3‐I ratio (Figure 3D), suggesting the activation of autophagy as a direct consequence of IGF2BP2 knockdown. The analysis of p62 expression in patients with TCGA‐LGG revealed that heightened p62 expression is associated with reduced overall survival (Figure 3E), higher glioma grades (Figure 3F), and increased prevalence in IDH wild‐type status (Figure 3G). Decreased p62 levels are often associated with increased autophagic activity.^40^ Associations between the expression level of p62 and clinical features in LGG enlightened a potential role of autophagy in the malignant development of glioma. Moreover, the CCK8 assay results demonstrated that the reduced cell viability resulting from IGF2BP2 knockdown could be restored when treated with autophagy inhibitors 3‐MA or HCQ (Figure 3H). These findings strongly suggest that the observed effects of IGF2BP2 knockdown in glioma cells may be attributed to its role in modulating autophagy. Additionally, the relationship between IGF2BP2 and autophagy was further substantiated through a mIHC assay performed on a TMA containing tumor samples, which revealed a significant positive correlation between IGF2BP2 and p62 expression (Figure 4), suggesting that IGF2BP2 is clinically relevant in modulating autophagy during glioma progression.

### 3.4 Correlation between IGF2BP2 expression and drug resistance

**Figure 5:**
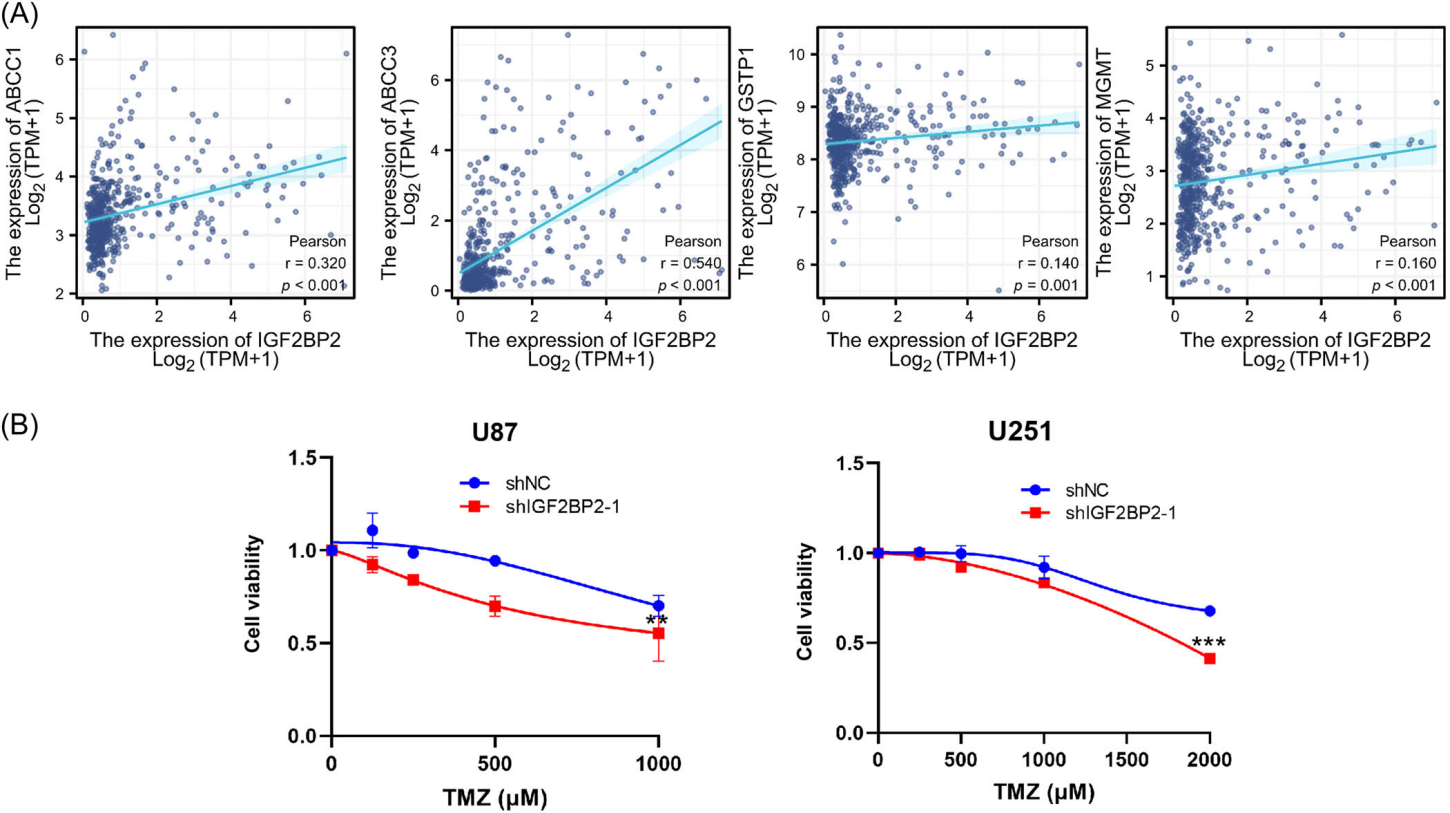
Insulin‐like growth factor 2 messenger RNA‐binding protein 2 (IGF2BP2) exhibited a correlation with drug‐resistant markers in TCGA‐low‐grade glioma (LGG) and its knockdown rendered glioma cells more susceptible to treatment with temozolomide (TMZ). (A) Correlation between IGF2BP2 and drug‐resistant markers (ABCC1, ABCC3, GSTP1, or MGMT) messenger RNA expression levels in TCGA‐LGG samples. Pearson correlation was conducted to analyze the correlation. (B) Cell Counting Kit‐8 assays were conducted to assess cell viability in U87 and U251 cells, with or without IGF2BP2 knockdown, following treatment with various concentrations of TMZ. Data are presented as the mean ± standard deviation (SD) from three independent experiments. ***p < 0.001, **p < 0.01, *p < 0.05. TCGA, The Cancer Genome Atlas. [Color figure can be viewed at wileyonlinelibrary.com]

To investigate the potential of IGF2BP2 as a therapeutic target for glioma, a correlation analysis employing TCGA‐LGG samples was conducted. The results revealed a positive correlation between IGF2BP2 mRNA expression and key drug‐resistant markers, including ABCC1,^41^, ^42^ ABCC3,^43^, ^44^ GSPT1,^45^ and MGMT^46^ (Figure 5A), which implied the connection between IGF2BP2 expression and drug resistance within glioma. TMZ is one of the most used drugs in glioma chemotherapy. The inhibition of autophagy was found to suppress the antitumor effect of TMZ.^26^ Based on these findings, we proposed that knocking down IGF2BP2, which induces cell autophagy, could potentially enhance the sensitivity of glioma cells to TMZ. The CCK8 assays demonstrated that U87 and U251 cells were more sensitive to TMZ treatment after IGF2BP2 knockdown (Figure 5B). This observed enhancement in treatment sensitivity signifies the intricate role of IGF2BP2 in modulating the cellular response to TMZ and making it a potential target for overcoming TMZ resistance.

## 4 DISCUSSION

Emerging evidence suggests that IGF2BP2 is involved in various cellular processes, including mRNA transport, stability, and localization. Several studies have revealed that IGF2BP2 plays a role in promoting glioma progression.^20^, ^22^ Nevertheless, the precise mechanism governing the impact of IGF2BP2 on glioma progression remains elusive. This study provides evidence for the significant role of IGF2BP2 in the regulation of cell viability, cell cycle progression, and colony‐formation in glioma cells, which suggests that IGF2BP2 plays a pivotal role in supporting cell survival and growth. Autophagy, a conserved cellular process, plays a dual role in cancer, acting as both a pro‐survival mechanism and a tumor suppressor.^25^, ^27^, ^47^ Autophagy can indeed promote cell survival under certain conditions, but it can also lead to cell death, referred to as autophagic cell death, especially when excessive or dysregulated.^47^, ^48^, ^49^ An intriguing aspect of this study is the identification of autophagy modulation as a potential mechanism underlying the observed effects on cell growth following IGF2BP2 knockdown. In this study, the rescue of decreased cell viability upon treatment with autophagy inhibitors, 3‐MA and HCQ, provides evidence for the involvement of autophagy in mediating the observed consequences of IGF2BP2 knockdown in glioma cells and implies that IGF2BP2's influence on glioma cell survival might be mediated through prevention of excessive autophagy. As we navigate the intricate balance between autophagy and cell survival, modulating IGF2BP2 activity could emerge as an avenue for influencing cancer cell behavior.

The observed decrease in p62 expression upon IGF2BP2 knockdown suggests a potential link between IGF2BP2 and p62 turnover. p62, also known as sequestosome 1, serves as a selective autophagy receptor that targets ubiquitinated substrates to autophagosomes for degradation.^50^ In this study, the reduction in p62 levels could indicate enhanced degradation, possibly through autophagy, as supported by the concomitant increase in LC3‐II level. LC3‐II is a hallmark of autophagosome formation.^51^ The elevation of LC3‐II further supports the notion that IGF2BP2 knockdown induces autophagy. The correlation between IGF2BP2 and p62 expression levels in tumor samples of patients with glioma further extends our in vitro findings. The TMA analysis provides clinical relevance to our observations, suggesting that the IGF2BP2‐p62 axis play a role in glioma pathogenesis. This opens avenues for further research into diagnostic and therapeutic strategies that target this axis. As we continue to unravel the mechanisms by which IGF2BP2 influences autophagy, the potential for developing targeted therapies in glioma treatment becomes increasingly promising.

Our findings not only contribute to understanding the intricate relationship between IGF2BP2, autophagy, and glioma progression but also hold potential biological significance. The fine‐tuned control exerted by IGF2BP2 on autophagy adds an extra layer of complexity to our understanding of glioma pathophysiology. The mechanism of how IGF2BP2 affects autophagy in glioma cells are intriguing and require further investigation. IGF2BP2, known as an RNA‐binding protein, may modulate autophagy through its interactions with specific RNA targets. Identifying these targets and understanding the pathways through which IGF2BP2 influences autophagy can provide critical insights into the regulatory mechanisms.

The involvement of IGF2BP2 in RNA recognition and posttranscriptional gene regulation makes it an intriguing target for therapeutic intervention. Modulating IGF2BP2 activity could potentially impact cancer cell behavior and open avenues for developing novel treatment strategies. The results presented in this study suggest that IGF2BP2 plays a role in glioma cell survival and autophagy regulation. Our study underscores the intricate relationship between IGF2BP2, autophagy, and glioma progression. These findings have potential implications for therapeutic interventions targeting autophagy in gliomas. Further investigations are warranted to unravel the precise molecular mechanisms by which IGF2BP2 influences autophagy and to explore its potential as a therapeutic target in glioma treatment, as our study revealed that knockdown of IGF2BP2 can sensitize TMZ to advance the autophagy of cancer cells.

## 5 CONCLUSIONS

IGF2BP2 plays a critical role in glioma progression, and knockdown of IGF2BP2 leads to cell autophagy, TMZ resensitization, and tumor suppression, making it a potential novel therapeutic target for glioma.

## AUTHOR CONTRIBUTIONS

Limei Deng was responsible for original draft, conceptualization, validation, formal analysis, and visualization. Ning Li contributed to review, edit, resources collection, and supervision. Yuming Zhang and Xilian Tang were responsible for writing, reviewing, and editing. Bingxi Lei contributed to writing, reviewing, editing, resources, and supervision. Qingyu Zhang was involved in writing, reviewing, editing, conceptualization, validation, formal analysis, visualization, resources, supervision, and funding acquisition. All authors read and approved the final manuscript.

## CONFLICT OF INTEREST STATEMENT

The authors declare no conflict of interest.

## ETHICS STATEMENT

The study involved the analysis of tumor samples obtained from 60 patients with clinical glioma who were treated at Sun Yat‐Sen Memorial Hospital, Sun Yat‐Sen University. The utilization of human glioma tumor TMA in line with the principles of China's Regulations on Human Genetic Resource Management. Approval was granted by the Medical Ethics Committee of Sun Yat‐sen Memorial Hospital, Sun Yat‐sen University (protocol: SYSKY‐2023‐108‐01). Informed consent was obtained from all participants or their legally authorized representatives. Before any data or sample collection, all patients or their legally authorized representatives were informed about the nature, purpose, risks, and benefits of the study. They were provided with detailed information regarding the study, including the procedures involved and the potential implications. Patients were made aware that participation in the study was entirely voluntary. They were not obliged to participate, and their decision had no impact on their medical care or relationship with Sun Yat‐Sen Memorial Hospital, Sun Yat‐Sen University. Patients were assured that their personal and medical information would be kept confidential and would not be disclosed in any publications arising from the study. Patients were informed that they had the right to withdraw their participation at any point without any repercussions, and this would not affect their medical treatment. All procedures involving human subjects in our study were performed in accordance with the ethical standards as laid down in the 1964 Declaration of Helsinki and its later amendments or comparable ethical standards.

## Data Availability

The data sets generated during and/or analyzed during the current study are available from the corresponding author on reasonable request.
